# Bilateral adrenal giant medullary lipoma combined with disorders of sex development: a rare case report and literature review

**DOI:** 10.3389/fonc.2023.1210679

**Published:** 2023-08-23

**Authors:** Chenghao Zhanghuang, Na Long, Zhen Yang, Yucheng Xie

**Affiliations:** ^1^ Department of Urology, Kunming Children’s Hospital, Yunnan Province Clinical Research Center for Children’s Health and Disease, Kunming, China; ^2^ Yunnan Key Laboratory of Children’s Major Disease Research, Yunnan Clinical Medical Center for Pediatric Diseases, Kunming Children’s Hospital, Kunming, China; ^3^ Special Ward, Kunming Children’s Hospital, Yunnan Province Clinical Research Center for Children’s Health and Disease, Kunming, China; ^4^ Department of Oncology, Yunnan Children Solid Tumor Treatment Center, Kunming Children’s Hospital, Kunming, China; ^5^ Department of Pathology, Kunming Children’s Hospital, Kunming, China; ^6^ Department of Pathology, The Second People’s Hospital of Yunnan Province, Kunming, China

**Keywords:** adrenal myelolipoma, adrenal gland, disorders of sex development (DSD), diagnosis, pathology

## Abstract

Bilateral adrenal myelolipoma is rare in clinics and patients with disorders of sex development (DSDs). One case was reported in our center. A 45-year-old patient was admitted to the hospital after discovering a left abdominal mass for more than a year and worsening abdominal pain for 18 days. An imaging examination showed bilateral adrenal masses. Physical examination showed clitoris hypertrophy with patelliform changes, thick and dense pubic hair, normal development of bilateral labia majora without labia minora, and urethral opening. After the relevant preoperative examinations, bilateral adrenal mass resection was performed under general anesthesia. The postoperative pathology confirmed adrenal myelolipoma. The incision healed well without recurrence over 10 years after the operation. Her enlarged clitoris decreased in size. This case report has a detailed diagnosis and treatment process and sufficient examination results. It can provide a reference for diagnosing and treating patients with bilateral adrenal myelolipoma and DSD and reduce the risk of misdiagnosis and mistreatment.

## Background

Myelolipoma is a rare non-functional benign tumor formed by the proliferation of mature adipose tissue and hematopoietic components of bone marrow. It was first described histologically by Gierke in 1905 and named myelolipoma by Oberling in 1929 (1). The incidence rate is 0.08%–0.2% (2); clinically, most are asymptomatic, whereas large tumors will cause waist and abdominal pain or rupture and bleeding, etc. In general, there is no endocrine function, and hypertension can also occur (3). Bilateral giant adrenal myelolipoma is rare. A case of bilateral adrenal giant myelolipoma with disorders of sex development (DSDs) was treated in our center, and the following is reported.

## Case report

The patient was 45 years old, and the social gender was female. The patient was admitted to the hospital after discovering a left abdominal mass for over a year and aggravation of abdominal pain for 18 days. In January 2008, the patient developed abdominal pain and discomfort without obvious causes. A left abdominal mass, about 4 cm × 3.5 cm × 3 cm in size, was found in the local hospital. In January 2009, the patient touched the left abdomen while bathing and felt that the mass was enlarged and hard, and abdominal pain recurred simultaneously. She was admitted to our hospital with a left abdominal mass of unknown etiology in February 2009. The patient had no fever, night sweats, jaundice, or emaciation. She had no menses since puberty. There was no family history.

Physical examination revealed a temperature of 37.2°C, respiration of 18 beats per minute, pulse of 72 beats per minute, and blood pressure of 120/80 mmHg. The patient had a body weight of 52 kg and a male face with visible whiskers and throat segments and a coarse, deep voice. The axillary hair was thick and dense, the chest was symmetrical without deformity, the breasts were not developed, and a physical examination of the heart and lungs showed no positive signs. The abdomen was soft with no palpable liver. However, a large mass was palpable in the left abdomen with a slightly hard texture and poor mobility, and there was no percussion pain in both renal region. The vulva showed hermaphroditism, thick and dense pubic hair, normal development of bilateral labia majora, no labia minora, hypertrophy of the clitoris like a short penis (4 cm long), no opening at the top, the urethral opening in the vestibule below the clitoris, and no vagina. On digital rectal examination, there was no palpable uterus or prostate-like tissue, no palpable mass in the pelvic cavity, and no testis-like tissue in the inguinal canal area: scoliosis deformity and free movement of limbs.

**Figure 1:**
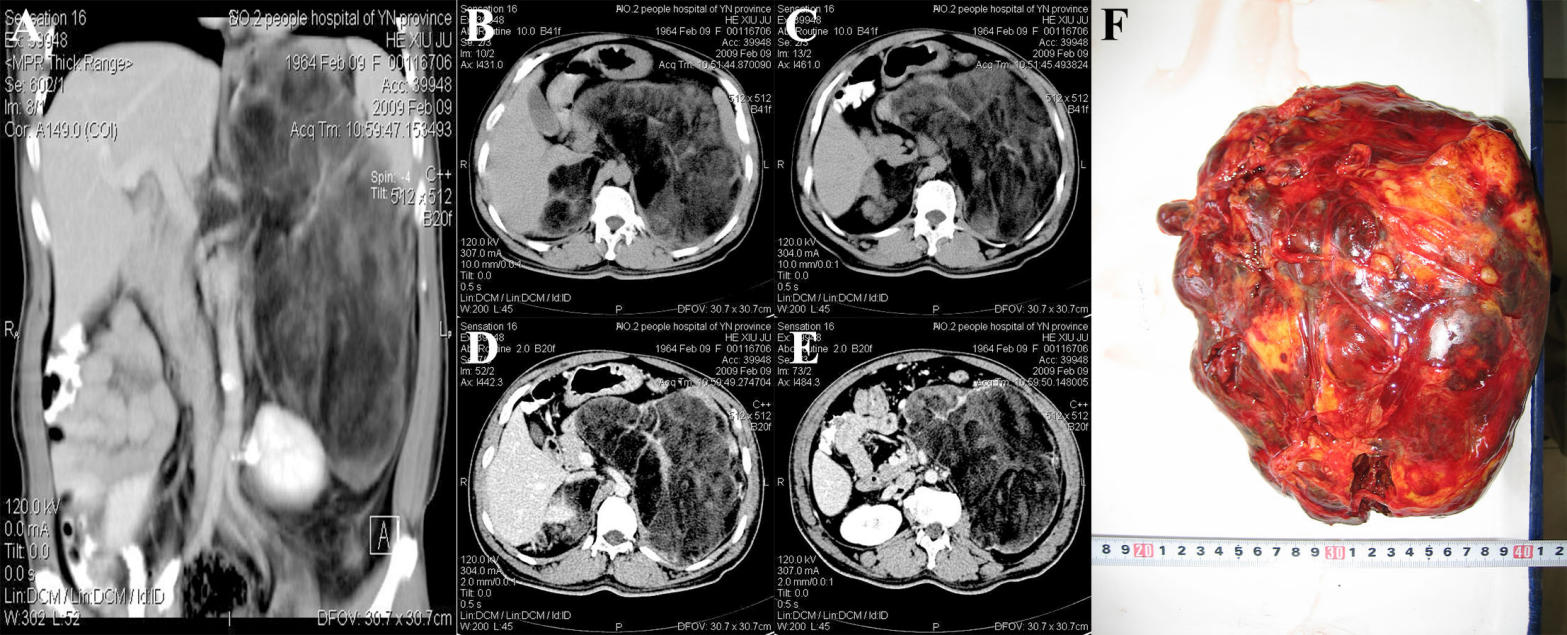
CT and gross specimens of the patient. **(A)** The left retroperitoneal mass was huge, and the left kidney was compressed and moved down. No enhancement was found in contrast-enhanced scan. **(B, C)** CT plain scan showed bilateral inhomogeneous density mass. **(D, E)** Bilateral adrenal enhanced CT findings. **(F)** Intraoperative gross specimen of a huge mass in the left adrenal gland.

Abdominal CT examination showed a huge mixed-density lesion (18 cm × 15 cm) in the left retroperitoneal space, including many irregular fat density shadows. The upper boundary of the mass was below the diaphragm, and the lower boundary was at the level of the iliac crest. The pancreas, stomach, and spleen were all compressed and displaced anteriorly, and the left kidney was compressed into the pelvis. A mixed-density shadow of 8 cm × 4 cm with clear borders was seen above the right kidney. On enhancement, the parenchymal portion of the left retroperitoneal mass showed variable enhancement that appeared to be encapsulated (**Figures 1A–E**). Abdominal and pelvic ultrasound showed a mixed echogenic mass in the left adrenal gland and a solid mass in the right upper abdomen. No definite sonograms of the uterus, prostate, or testis were found. Laboratory tests showed white blood cells of 4.39 × 10^9^/L, neutrophils of 74.7%, lymphocytes decreased by 13.9%, and monocytes increased by 10.5%. The red blood cell count was 5.30 × 10^12^/L, and the hemoglobin was 177 g/L. Total bilirubin, direct bilirubin, and indirect bilirubin increased to 30, 11.3, and 18.7 μmol/L, respectively. Total cholesterol decreased to 2.55 mmol/L, and high-density lipoprotein decreased to 0.95 mmol/L. Other parameters, such as blood glucose and renal function, were normal. Tumor markers such as alpha-fetoprotein, human chorionic gonadotropin, neuron-specific enolase, and carcinoembryonic antigen were negative. Karyotype analysis of the patient’s peripheral blood showed that the patient was 46, XX.

The patient underwent open bilateral adrenalectomy under general anesthesia. During the operation, bilateral subcostal transverse incisions were made, the liver and transverse colon were pulled away to expose the retroperitoneal space, and the left retroperitoneal space was dissected after careful mobilization. There was a solid cystic mass (about 24 cm × 16 cm in size), dark, and hard in texture. The mass was dissected carefully to avoid injury to the renal vein. The huge mass was completely removed, and the other side was about 9 cm × 5 cm removed. A part of the bilateral mass after complete resection was sent for intraoperative rapid frozen section diagnosis. Intraoperative frozen report: The tumor was derived from adipose tissue and tended to be benign. Lymph node dissection was not performed. After sufficient hemostasis of the operative field, a drainage tube was placed, and the abdominal incision was sutured layer by layer. The intraoperative blood loss was about 500 mL. The operation time was 3 h and 15 min. The operation was uneventful, and anesthesia was stable without a blood transfusion. The patient returned to the ward safely after the operation. The drainage tube was removed 7 days after the operation, and the incision healed well 14 days after the suture was removed. The patient was discharged.

Pathological examination: gross observation: The left side was a yellow-red or dark-red mass, weighing 2.3 kg, measuring 24 cm × 18 cm × 10 cm, with a complete capsule, solid cut surface, and pale yellow fat mixed with reddish-brown (**Figure 1F**). The right side was a yellow mass measuring 8.5 cm × 4.3 cm × 2.8 cm with an intact capsule and fatty cut surface. Microscopic examination showed that the left tumor mainly showed erythroid, granulocytic, and megakaryocytic bone marrow hematopoietic cells without atypia. The erythroid lineage was dominated by mid- and late-stage erythrocytes, whereas the granulocyte was dominated by mid- and late-young rod and lobulated nuclei. Lymphocytes were scattered or aggregated, and megakaryocytes were scattered, one to three per high-power field. Hemosiderin phagocytosed histiocytes and slight hyperplasia of fibrous tissue were seen locally. A large amount of mature adipose tissue was intermingled. At the edge of the tumor, adrenal globular zone cell clusters of different sizes were seen, and massive hemorrhage was seen in some areas. On the right side, the tumor mainly comprised mature adipose tissue mixed with bone marrow hematopoietic tissue. Under the tumor capsule, the adrenal zona glomerular cells were rarely compressed. The pathological diagnosis was bilateral adrenal myelolipoma with hemorrhage (the left tumor was huge). The adrenal myelolipoma was considered a benign tumor without secretory function, and no chemotherapy, radiotherapy, or endocrine therapy was given after the operation. The patient was followed up for 14 years and 2 months, and the general condition was good, without discomfort and tumor recurrence.

## Discussion

**Figure 2:**
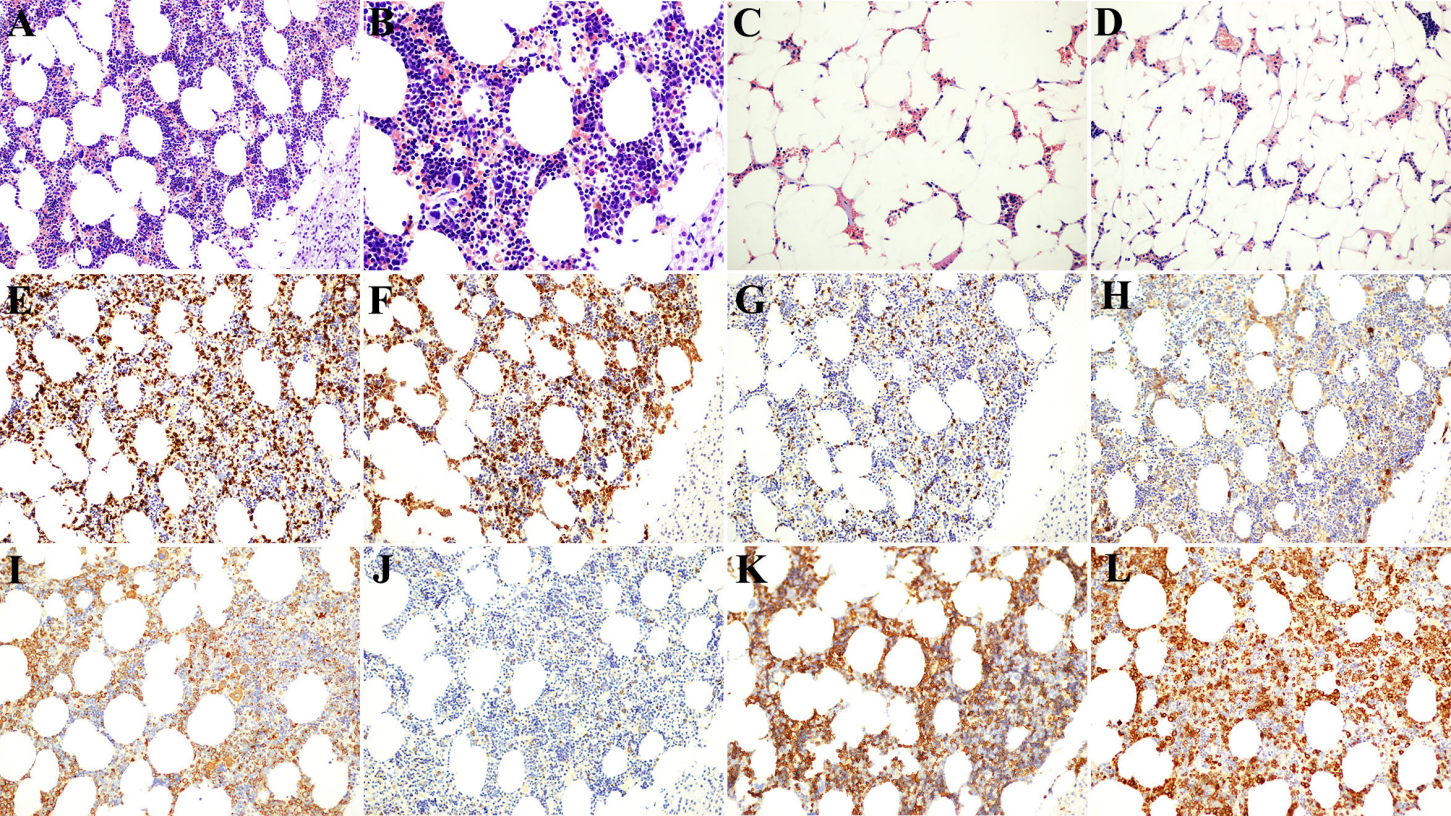
Postoperative pathological and immunohistochemical results. **(A, B)** The left tumor was composed of fat cells and various hematopoietic cells, and the lower right was the residual adrenal cortex [Immunohistochemistry (HE); **(A)** 200× and **(B)** 400×]. **(C, D)** The right mass was dominated by adipocytes, with scattered small foci of hematopoietic cells (HE; 200×). **(E)** CD3-positive T lymphocytes (200×). **(F)** CD15-positive granulocytes (200×). **(G)** CD20-positive B lymphocytes (200×). **(H)** CD38-positive plasma cells (200×). **(I)** CD63-positive monocytes and plate-producing cells (200×). **(J)** CD117-positive mast cells (200×). **(K)** CD235a-positive nucleated erythrocytes and mature erythrocytes (200×). **(L)** Myeloperoxidase (MPO)-positive myeloid cells (200×).

The myelolipoma was composed of a mixture of mature fat and bone marrow in varying proportions. In this case, the multifocal dark red area on the left side was dominated by bone marrow hematopoietic tissue, and a few residual zona glomerulus cells were found under the tumor capsule (**Figures 2A, B**). The right side was dominated by adipose tissue, and only a little bone marrow hematopoietic tissue was scattered among adipocytes (**Figures 2C, D**). Myelolipoma is more common in the adrenal gland than in the thoracic cavity, retroperitoneum, presacral area (4), mediastinum, spleen, lung, testis, soft tissue, and other parts. It has been reported that (5) occurs simultaneously in the adrenal gland and the contralateral pelvic cavity, and it is extremely rare that the primary tumor occurs in the liver (6, 7). The most common age of adrenal myelolipoma was 50 to 70, and most patients were adults. The incidence of male and female patients was roughly equal. Most tumors are solitary, with a slightly higher incidence on the right side than on the left side and rare on both sides (8). There were no atypia or lip blasts in the adipocytes. Hematopoietic tissue was sparsely or widely distributed, and lymphocytes were scattered or aggregated. Immunohistochemical markers showed granulocytic, erythroid, lymphoid, and megakaryocytic hematopoietic cells at various stages of differentiation (**Figures 2E–L**).

Differential diagnosis with the following tumors is required: 1. The common type of lipoma has no hematopoietic component, which differs from the location of myelolipoma. It should be noted that some myelolipomas may have fewer hematopoietic components and can be misdiagnosed as lipomas. 2. For well-differentiated liposarcoma, overlapping with myelolipoma but without hematopoietic components, a careful search for immunohistochemical markers can help. Atypical stromal cells with hyperchromatic chromatin or lip blasts can be found. 3. Myeloid neoplasms, often multiple lesions, accompanied by the liver and spleen enlargement and blood system abnormalities. The tumor showed diffuse infiltration of primitive tumor cells, which were atypical and monomorphic. Immunohistochemical staining showed the absence of normal multilineage hematopoietic components of bone marrow. 4. Extramedullary hematopoiesis, usually in the liver, spleen, and lymph nodes, is associated with lymphohematopoietic disease, and the lesions are often multifocal rather than isolated, well-defined nodules.

Endocrine dysfunction can promote the occurrence of adrenal myelolipoma. Statistics show that about 10% of myelolipoma cases are complicated by endocrine insufficiency, including Cushing syndrome, primary aldosteronism, congenital adrenal hyperplasia (CAH), and hyperparathyroidism (9). CAH is an autosomal negative genetic disease caused by defects in adrenocortical hormone synthase, such as 21-hydroxylase, 17-hydroxylase, or 11-hydroxylase (10). Because of the disorder of the glucocorticoid synthesis pathway, such diseases can lead to obvious hyperandrogenism and hyper-adrenocorticotropic hormone (11). According to statistics, the incidence of adrenal myelolipoma in patients with CAH is significantly higher than that in normal people (about 6%). Its incidence is positively correlated with the level of serum adrenocorticotropic hormone (12). Cases of adrenal myelolipoma with obvious hyperandrogenism have also been reported in the literature (13). Therefore, the pathogenesis of adrenal myelolipoma is closely related to the overexpression of androgen and adrenocorticotropic hormone. The patient was diagnosed with male pseudohermaphroditism, accompanied by amenorrhea and clitoral hypertrophy after puberty, which was considered to be related to the excessive expression of androgens caused by adrenal myelolipoma. The symptoms of clitoral hypertrophy were relieved after surgical resection.

There are many studies on the nature of myelolipomas. Bishop et al. (9) found that most myelolipomas have non-random X chromosome inactivation, suggesting that myelolipomas are of monoclonal origin and belong to genuine tumors. The etiology and pathogenesis of adrenal myelolipoma are still unclear. They may be related to adrenal cortical metaplasia induced by necrosis, infection, stress, and other factors (14–16). Cytogenetic examination of adrenal myelolipoma has been reported with chromosome (3; 21) (q25; 11) translocation, which also suggests a true tumor (17). Some authors point out that adrenal myelolipoma often occurs in endocrine diseases or chronic wasting diseases, which may stimulate the differentiation of adrenal cortical mesenchymal cells into myelocytes or adipocytes (18). At present, the presence of bone tissue in myelolipoma is controversial, and most scholars state that bone tissue is metaplasia.

Myelolipoma is generally asymptomatic, with a volume of less than 5 cm and rarely larger than 10 cm (19). The large volume can cause abdominal distension, pain, or other compression symptoms. There are reports of giant adrenal myelolipoma with a maximum diameter of 15–16 cm (20). In this case, the patient had myelolipoma in both adrenal glands, and the left tumor was huge, with a maximum diameter of 24 cm, accompanied by DSD, manifested as clitoral hypertrophy and no vagina. The principle of myelolipoma treatment is small, and asymptomatic can be conservative follow-up. Yalagachin et al. (21) considered that adrenal myelolipoma with function or diameter ≥6 cm should be treated with adrenal tumor resection, and surgical resection of huge tumors can relieve symptoms and prevent complications such as bleeding and rupture. In the past, open surgery was the main clinical operation for giant myelolipoma. However, with the development of laparoscopic technology, laparoscopic adrenalectomy has become the first choice for adults. Of note, is the transabdominal or retroperitoneal approach more advantageous? It is still controversial (22). In recent years, good results of robotic surgical treatment have also been reported (23). In addition, studies have shown that minimally invasive surgery can effectively reduce surgical site infection caused by open surgery so that patients can obtain better perioperative outcomes (24).

## Conclusion

In conclusion, myelolipoma is rare in clinical practice, and it is rare to present a huge mass in both adrenal glands with DSD. Adrenal myelolipoma is a benign non-secretory tumor. The tumor was named for the presence of mature adipocytes and bone marrow cells. The disease may result from abnormal cortical reticular endothelial cell metaplasia development or aberrant embryonic residues. However, patients with adrenal myelolipoma that produce compression symptoms such as hypertension need surgical resection of the tumor to relieve the symptoms. Adrenal myelolipoma is considered a benign tumor without secretory function, and postoperative chemoradiotherapy and endocrine therapy are not needed. Patients with adrenal myelolipoma complicated with disorders of sexual development can be followed up after resection of adrenal myelolipoma before oculoplastic surgery, and some patients with disorders of sexual development can spontaneously relieve the abnormal manifestations of external genitalia.

## Data availability statement

The original contributions presented in the study are included in the article/supplementary material. Further inquiries can be directed to the corresponding author.

## Ethics statement

The studies involving human participants were reviewed and approved by the Ethical Committee of Kunming Children’s Hospital (2022-12-001-K01). This study is by the relevant guidelines and regulations. The data in this study were obtained from this patient and his legal guardian. Written informed consent was obtained from the patient’s parents.

## Author contributions

CZ designed the study. CZ, NL, and ZY collected and analyzed the data. CZ drafted the initial manuscript. CZ revised the article critically. CZ, ZY, NL, BY, and YX reviewed and edited the article. NL and CZ are co-first authors. All authors approved the final manuscript.
